# The Prevalence and Correlates of Lifetime Psychiatric Disorders and Trauma Exposures in Urban and Rural Settings: Results from the National Comorbidity Survey Replication (NCS-R)

**DOI:** 10.1371/journal.pone.0112416

**Published:** 2014-11-07

**Authors:** Jennifer S. McCall-Hosenfeld, Sucharita Mukherjee, Erik B. Lehman

**Affiliations:** 1 Department of Medicine, The Pennsylvania State University College of Medicine, Hershey, Pennsylvania, United States of America; 2 Department of Public Health Sciences, The Pennsylvania State University College of Medicine, Hershey, Pennsylvania, United States of America; 3 Department of Pediatrics, University of Rochester Medical Center, Rochester, New York, United States of America; Univ of Toledo, United States of America

## Abstract

**Introduction:**

Distinctions between rural and urban environments produce different frequencies of traumatic exposures and psychiatric disorders. We examine the prevalence of psychiatric disorders and frequency of trauma exposures by position on the rural-urban continuum.

**Methods:**

The National Comorbidity Survey Replication (NCS-R) was used to evaluate psychiatric disorders among a nationally-representative sample of the U.S. population. Rurality was designated using the Department of Agriculture's 2003 rural-urban continuum codes (RUCC), which differentiate counties into levels of rurality by population density and adjacency to metropolitan areas. Lifetime psychiatric disorders included post-traumatic stress disorder (PTSD), anxiety disorders, major depressive disorder, mood disorders, impulse-control disorders, and substance abuse. Trauma exposures were classified as war-related, accident-related, disaster-related, interpersonal or other. Weighted logistic regression models examined the odds of psychiatric disorders and trauma exposures by position on the rural-urban continuum, adjusted for relevant covariates.

**Results:**

75% of participants were metropolitan, 12.2% were suburban, and 12.8% were from rural counties. The most common disorder reported was any anxiety disorder (38.5%). Drug abuse was more common among metropolitan (8.7%, p = 0.018), compared to nonmetropolitan (5.1% suburban, 6.1% rural) participants. A one-category increase in rurality was associated with decreased odds for war-related trauma (aOR = 0.86, 95%CI 0.78–0.95). Rurality was not associated with risk for any other lifetime psychiatric disorders or trauma exposure.

**Discussion/Conclusions:**

Contrary to the expectation of some rural primary care providers, the frequencies of most psychiatric disorders and trauma exposures are similar across the rural-urban continuum, reinforcing calls to improve mental healthcare access in resource-poor rural communities.

## Introduction

Psychiatric disorders are extremely common in the United States. Every year, approximately one in four Americans (26.2%) suffers from a diagnosable mental illness [1]. About half (46.4%) of the U.S. population experiences a psychiatric disorder diagnosable by the Diagnostic and Statistical Manual, 4^th^ edition (DSM-IV), the standard tool for assessing psychiatric disorders, at some point in their lives [1]. The overall cost of mental illness to the U.S. economy is staggering, with a 2008 report estimating costs over $300 billion, comprised of both the direct costs of mental health care, as well as indirect costs including loss of income from unemployment [2].

While mental health issues represent a significant problem, causes of mental illnesses are complex. However, both genetic liability and environmental factors have been shown to play a crucial role in mental health outcomes [3]. Exposure to trauma is one such environmental factor that is associated with the development of psychiatric illness [4]. Fifty-one percent of women and 60% of men experience at least one traumatic event in their lives [5]. Although not all trauma-exposed individuals develop a psychiatric disorder [6], trauma history is implicated in the etiology of posttraumatic stress disorder (PTSD) [4], major depression [4], [7]–[8], and substance use disorders [9].

Because the development of mental illnesses is impacted by environmental factors, it is important to consider whether different environments – such as position on the rural-urban continuum – produce different frequencies of risk factors associated with mental health outcomes. Position on the rural-urban continuum is associated with numerous sociodemographic factors which have been shown to affect the frequency of mental illnesses. For example, residents of rural communities have lower incomes and educational status compared to non-rural individuals and are more likely to be uninsured [10]–[11]. These differing population characteristics likely play a role in the increased frequency of mental illness such as depression which is sometimes noted among rural residents [12]. Socidemographic disparities affecting rural residents, and the resultant impact on mental health, are particularly important to consider in light of the fact that rural residing individuals face substantial service deficits related to healthcare, especially mental healthcare. Approximately 20% of non-metropolitian counties lack adequate mental health services compared to 5% of metropolitan counties [13].

One previously unexplored, but possibly important factor that may contribute to rural-urban mental health disparities is differences in the frequency of trauma exposures across the rural-urban continuum. Some rural healthcare providers have suggested that rural patients are exposed to fewer traumas compared to non-rural patients [14], suggesting that distinctions between rural and urban environments may produce different frequencies of traumatic exposures. However, little prior work has explicitly examined whether trauma exposures truly differ across the rural-urban continuum.

Although some prior studies have examined rural-urban disparities in mental health, most prior studies have not used a nationally representative dataset [15]–[19]. Given that many of these studies were not completed using national data or focused on specific populations, there is variation among the results. One study using a North Carolina population reported major depressive disorders as being twice as frequent in urban areas [17], with another study focusing on Mexican-Americans in California also reporting a higher prevalence of any psychiatric disorder in urban areas [15]. In contrast, a study evaluating cancer survivors found more symptoms of anxiety and depression among rural populations [16], and a study done in the Midwest found substance use disorders more prevalent among rural residents [18]. Another study found the prevalence of psychiatric disorders in rural primary care offices to be as high as those in urban offices [19]. At least one prior study examined frequency of depression comparing rural and urban residents in a national sample and found the unadjusted prevalence of depression to be slightly higher among rural residents [12], but this difference disappeared once covariates were adjusted. Other U.S. studies found the prevalence to be equivalent across various geographic indicators such as region and rurality [20], [21].

Internationally, a meta-analysis of urban-rural differences showed a higher prevalence of any disorder, mood disorders and anxiety disorders in urban areas compared to rural areas. However, this study included many international sites and the articles comprising the meta-analysis were heterogeneous [22]. A more recent, nationally representative study performed using a novel indicator of rurality found that, among adults, risk for major depression and serious mental illness was slightly elevated in intermediate categories of rurality – i.e., small metro and semi-rural areas – compared to large metropolitan areas and fully rural areas [23]. Thus, the data examining mental health across the rural-urban continuum has not conclusively shown a rural-urban disparity, and may not even reflect a linear relationship.

To remediate these knowledge deficits, we used the National Comorbidity Survey Replication (NCS-R) to examine the frequency of lifetime psychiatric disorders and trauma exposures across the urban-rural continuum. The primary objective of this study was to examine the association between position on the rural-urban continuum and both trauma exposures and lifetime psychiatric disorders, and to determine whether these variables differ by degree of rurality.

## Materials and Methods

### Study population and data collection

The NCS-R is a nationally representative community household survey that measured the correlates and prevalence of psychiatric disorders in the United States. The NCS-R is representative of the U.S. population based on a variety of census indicators. The population under study included people at least 18 years of age, who were interviewed in-person between February 2001 and April 2003 [24]. Data from the NCS-R includes assessments of several psychiatric disorders, such as PTSD, depression and anxiety as well as self-reports of abuse and other traumas [24].

### Ethics statement

Prior to subject involvement in the NCS-R, the human subject participant committees from both the Harvard Medical School and the University of Michigan provided ethical review [21]. Interviewers explained the study and obtained verbal informed consent prior to beginning each interview. Recruitment and consent were approved by the Human Subjects Committees of Harvard Medical School, Boston, Massachusetts, and the University of Michigan. The Pennsylvania State University College of Medicine Institutional Review Board (IRB) reviewed and approved this secondary analysis.

The NCS-R assessed a wide range of DSM-IV psychiatric disorders through structured interviews administered in two different parts [24]. The first part of the interview included core diagnostic assessments completed by all the participants (*n = 9,282*). The Part II interview focused on participants with potential for psychiatric disorders (*n = 5,692*) [24]. The assessment of lifetime PTSD, trauma exposure, and other relevant health conditions were all included in Part II. The overall response rate was 70.9% for the primary interview and 80.4% among pre-designated secondary participants [24]. A detailed description of the interview schedule, survey population, fieldwork, organization and procedures, weighting and sample design used in the NCS-R has been previously published [24]. As noted above, the goal of the current analysis is an assessment of frequencies of specific mental health diagnoses and trauma exposures. Data for these exposures and diagnoses were only included in the Part II questionnaire. Thus, only participants who answered Part II of the NCS-R are included in these analyses, with appropriate sample weighting applied.

### Measures

#### Rural-urban continuum

The primary unit of analysis in this study is the study participant's geographic placement on the rural-urban continuum. Rurality/urbanicity was designated based on county of residence using the Department of Agriculture's 2003 rural-urban continuum codes (RUCCs). The 2003 rural-urban continuum is a categorization scheme that differentiates metropolitan from nonmetropolitan counties by population size, degree of urbanization and functional proximity (adjacency) to metropolitan areas [25]. Using this classification, county codes range from 1 (most urban) to 9 (most rural). Federal Information Processing Standard (FIPS) county identifiers were used to match the participant's county of residence to RUCC. In the NCS-R data, no individuals from the Part II data were included from counties with RUCCs 8-9.

#### Outcome: Traumatic event exposure history

Participants were asked about their history of over 27 specific traumas and could also report any other additional traumas that were not pre-specified. The trauma variables were further divided into five major trauma categories, based upon a scheme previously developed by Nickerson et al. [26]: War-related, Accident-related, Disaster-related, Interpersonal and Other traumas. War-related trauma was defined as having combat experience, peacekeeping, being an unarmed civilian in war, being a civilian in ongoing terror, or being a refugee. Accident-related trauma was defined as being exposed to a toxic chemical, being involved in a life-threatening motor-vehicle accident (MVA), or being in another life-threatening accident. Disaster-related trauma was defined as experiencing a major natural disaster or experiencing a man-made disaster. Interpersonal trauma was defined as being kidnapped or held captive, being badly beaten by parents, being badly beaten by a partner, being badly beaten by someone else, being mugged, held up or threatened with a weapon, being raped, being sexually assaulted, being stalked, or witnessing serious physical fights at home as a child, seeing someone badly injured/killed or unexpectedly seeing a dead body. Other trauma was defined as experiencing a life-threatening illness, having someone very close die unexpectedly, having a child with a life-threatening illness or injury, having someone close experience an extremely traumatic event, or seeing atrocities or carnage.

#### Outcome: Lifetime psychiatric disorders

Psychiatric disorders were assessed using the World Mental Health Survey Initiative version of the World Health Organization Composite International Diagnostic Interview (WMH-CIDI), which is a structured diagnostic interview from which DSM-IV diagnoses are derived [24]. The DSM-IV diagnoses considered in this study include the following: (1) posttraumatic stress disorder (PTSD), (2) Any anxiety disorder (panic disorder, generalized anxiety disorder, agoraphobia without panic disorder, specific phobia, social phobia, PTSD, separation anxiety disorder). (3) Major Depressive Disorder, (4) Any mood disorder (major depressive disorder, dysthymia, bipolar disorder I, bipolar disorder II), (5) Any impulse-control disorder (oppositional defiant disorder, conduct disorder, attention-deficit/hyperactivity disorder and intermittent explosive disorder), (6) Alcohol use disorders (alcohol abuse with or without dependence), (7) Drug use disorders (drug abuse with or without dependence), (8) Any substance use disorder (nicotine dependence, alcohol and drug abuse and dependence), (9) Any disorder (which includes any mood, anxiety, impulse control or substance use disorder previously mentioned).

#### Sociodemographic variables

The interview was comprised of an extensive demographic section, which assessed gender, age, race-ethnicity, household income, education, current employment, marital status, number of children and insurance coverage. Insurance coverage was further divided into none, private (health insurance obtained through employer/union, health insurance plan purchased from insurance company, health insurance not otherwise mentioned) or public (military health insurance, Medicare, government assistant program for people in need, state health insurance, and Indian health service).

### Statistical analysis

All statistical tests utilized appropriate weights to account for the complex sampling design of the NCS-R. Analyses were conducted via SAS Software version 9.3 (SAS Institute, Cary, NC). We first developed a three-level rurality variable, dividing RUCCs into categories based on existing USDA categorization [25]: metropolitan-urban counties (RUCC 1–3), nonmetropolitan counties with populations of 20,000+ (RUCC 4–5), and nonmetropolitan counties with populations less than 19,999 (RUCC 6–7). Weighted Chi-square tests were used to assess bivariate associations between county-level rural-urban continuum indicators and key demographic descriptors, mental health diagnoses and trauma exposures using the three-level rurality variable. We then assessed for multicollinearity among the candidate control variables (demographic factors) by examining variable inflation factor (VIF) statistics. Since no significant multicollinearity was found, all candidate control variables were then introduced into multivariable analyses.

To examine whether the associations between mental health conditions and trauma exposures differed by degree of rurality, a weighted multivariable logistic regression was used to determine the association of the 7-level ordinal rural-urban continuum variable with the various types of lifetime trauma exposure, controlling for all prespecified demographic factors. Separate models were developed for each identified mental health diagnosis. Likewise, this approach was used to examine the association of rural-urban continuum with mental disorders. Adjusted odds ratios were used to quantify the magnitude and direction of the associations for all logistic regression analyses.

To examine for threshold effects and to consider the possibility that a nonlinear relationship existed between position on the rural-urban continuum and both mental health diagnoses and trauma exposures, we performed post-hoc analyses in which we repeated the multivariable regressions, re-examining our data using both a three-level rurality variable (metropolitan/suburban/rural) and a two-level rurality variable (metropolitan versus all others.)

## Results

Table 1 shows the results of weighted bivariate analysis by three level rural-urban county category. About 75% of participants resided in major metropolitan counties (RUCC 1–3), 12.2% were from nonmetropolitan counties with an urban population of greater than 20,000 (RUCC 4–5), and 12.8% were from nonmetropolitan counties with an urban population of less than 20,000 (RUCC 6–7). Most self-identified as white (72.7%), were currently employed 64.8%), married (56%) and had private insurance coverage (73%). Participants from households with incomes less than <$19,999 (25.4% of the rural population vs. 20.7% of the metropolitan population, p = 0.007) and with lower educational attainment (10.6% of the rural population with college degree or more vs. 24.8% of the metropolitan population, p<.001) were more likely to reside in non-metropolitan counties.

**Table 1:** Characteristics of NCS-R participants by rurality classification*.

	Total		Major Metropolitan		Suburban Counties		Rural Counties		
	*N = 5692*	100.0%	*N = 4688*	75.0%	*N = 519*	12.2%	*N = 485*	12.8%	P-value
**Female**	3310	53.0	2723	52.8	309	56.0	278	51.7	0.188
**Race**									
Hispanic	527	11.1	479	12.0	37	12.1	11	4.8	0.538
Non-Hispanic Black	717	12.4	613	13.4	64	11.2	40	7.5	
Non-Hispanic White	4180	72.7	3392	70.3	371	73.3	417	86.8	
Other	268	3.8	204	4.4	47	3.4	17	1.0	
**Age [years]**									
18 to 29	1371	23.5	1109	23.8	155	25.2	107	20.1	0.320
30 to 44	1826	28.8	1547	29.9	139	26.4	140	25.2	
45 to 59	1521	26.5	1249	26.0	130	26.7	142	29.0	
60+	974	21.2	783	20.3	95	21.7	96	25.7	
**Household Income**									
0 to $19,999	1182	22.0	923	20.7	142	26.1	117	25.4	0.007
$20,000 to $34,999	921	15.9	740	15.9	95	16.5	86	15.3	
$35,000 to $69,999	1811	30.6	1475	29.8	159	32.0	177	34.4	
$70,000+	1778	31.5	1550	33.6	123	25.3	105	24.9	
**Education**									
0 to 11 years (less than high school)	849	16.8	647	15.2	90	18.9	112	24.0	<0.001
12 years (equivalent of high school)	1712	32.5	1379	31.2	133	28.4	200	44.6	
13 to 15 years (some college)	1709	27.5	1439	28.9	157	26.5	113	20.8	
16-plus years (college degree or more)	1422	23.2	1223	24.8	139	26.2	60	10.6	
**Current Employment**									
Employed	3766	64.8	3128	65.7	335	63.7	303	60.4	0.569
Unemployed	283	5.1	223	4.8	30	6.1	30	5.9	
Not in labor force	1630	30.1	1329	29.5	154	30.2	147	33.7	
**Marital Status**									
Married	3236	56.0	2618	54.5	295	56.3	323	63.9	0.101
Separated/Widowed/Divorced	1239	20.8	1041	21.1	105	20.7	93	19.1	
Never Married	1217	23.2	1029	24.3	119	23.0	69	17.0	
**Insurance Coverage**									
None	730	12.9	589	12.3	75	17.6	66	11.9	0.454
Private	4164	73.0	3451	73.6	367	69.4	346	73.5	
Public	782	14.1	636	14.1	75	13.0	71	14.6	
**Disorder**									
Posttraumatic Stress Disorder lifetime	600	6.8	502	6.8	54	6.9	44	7.0	0.986
Any Anxiety Disorder	3058	38.5	2518	38.1	283	38.4	257	40.5	0.925
Major Depressive Disorder lifetime	1548	16.6	1288	16.5	146	17.7	114	16.2	0.913
Any Mood Disorder	1781	19.1	1480	19.0	165	19.6	136	18.9	0.978
Any Impulse-Control Disorder	1244	15.4	1050	16.0	90	12.2	104	15.3	0.437
Alcohol Abuse	1027	13.2	867	13.5	79	12.4	81	12.2	0.860
Drug Abuse	651	8.0	570	8.7	37	5.1	44	6.1	0.018
Any Substance Abuse	1495	19.5	1261	19.8	119	18.4	115	19.2	0.956
Any Disorder	4155	53.5	3448	53.7	365	51.6	342	54.2	0.950
**Trauma**									
War-Related Trauma	530	9.5	469	10.5	34	6.0	27	7.0	0.021
Accident-Related Trauma	1823	29.4	1511	29.2	147	26.3	165	33.0	0.534
Disaster-Related Trauma	1309	21.8	1089	21.6	125	22.3	95	23.0	0.952
Interpersonal Trauma	3526	54.9	2934	55.0	294	52.6	298	56.6	0.834
Other Type of Trauma	3540	58.0	2924	57.9	299	52.3	317	64.1	0.025

*All analyses applied appropriate sample weights.

In our unadjusted analyses applying appropriate sample weights (Table 1), we found that both lifetime mental illnesses and trauma exposures were common, with a prevalence of>50% among all categories of rurality. The most common mental health disorder reported was any anxiety disorder (38.5%) followed by any substance use disorder (19.5%). Drug use disorders were more common for participants residing in metropolitan areas (8.7%) when compared to nonmetropolitan areas (5.1% for suburban, 6.1% for rural, p = 0.018). War-related trauma was more common for individuals from metropolitan areas (10.5%, versus 6.0% for suburban and 7.0% for rural, p = 0.021) and other types of trauma were more common for individuals from rural areas (64.1%, versus 52.3% for suburban and 57.9% for rural, p = 0.025).

Table 2 shows the results of weighted ordinal logistic regressions examining trauma exposures across the 7-level ordinal rural-urban continuum variable. A one-unit increase in rurality as indicated by a one-level increase in county RUCC was associated with a 14% decreased odds for war-related trauma (adjusted Odds Ratio (aOR)  = 0.86, 95% Confidence Interval (CI): 0.78–0.95). The remaining trauma exposure categories were not significantly associated with position on the urban-rural continuum.

**Table 2:** Adjusted odds ratios (aOR) for trauma exposure by rurality, NCS-R participants* †.

	War-related trauma	Accident-related trauma	Disaster-related trauma	Interpersonal trauma	Other trauma
	aOR (95% CI)	aOR (95% CI)	aOR (95% CI)	aOR (95% CI)	aOR (95% CI)
**Rurality** (1-unit increase)	0.86 (0.78, 0.95)	1.01 (0.94, 1.08)	1.01 (0.91, 1.13)	0.98 (0.91, 1.06)	1.00 (0.96, 1.06)
**Female** (vs. Male)	0.21 (0.16, 0.29)	0.40 (0.33, 0.49)	0.68 (0.56, 0.82)	0.73 (0.64, 0.83)	0.88 (0.75, 1.03)
**Race**					
Hispanic	0.86 (0.42, 1.75)	0.67 (0.53, 0.85)	1.13 (0.83, 1.54)	0.95 (0.66, 1.37)	0.50 (0.38, 0.64)
Non-Hispanic Black	1.16 (0.81, 1.67)	0.80 (0.65, 0.98)	0.89 (0.65, 1.21)	1.10 (0.83, 1.47)	0.83 (0.64, 1.07)
Non-Hispanic White	Reference	Reference	Reference	Reference	Reference
Other	1.40 (0.78, 2.52)	1.31 (0.80, 2.12)	1.28 (0.81, 2.02)	1.21 (0.73, 2.01)	1.06 (0.70, 1.62)
**Age**					
18 to 29	Reference	Reference	Reference	Reference	Reference
30 to 44	1.21 (0.75, 1.98)	1.02 (0.82, 1.28)	0.92 (0.70, 1.22)	1.08 (0.86, 1.36)	0.94 (0.75, 1.18)
45 to 59	2.49 (1.62, 3.85)	1.34 (1.04, 1.71)	1.22 (0.98, 1.52)	1.13 (0.93, 1.36)	1.42 (1.18, 1.70)
60+	3.35 (2.01, 5.61)	0.76 (0.56, 1.04)	1.10 (0.84, 1.45)	0.48 (0.38, 0.60)	1.22 (0.93, 1.60)
**Household Income**					
$0 to $19999	0.61 (0.36, 1.03)	1.04 (0.78, 1.39)	1.08 (0.85, 1.38)	1.01 (0.81, 1.25)	0.92 (0.71, 1.20)
$20000 to $34999	0.92 (0.66, 1.29)	1.02 (0.85, 1.23)	0.74 (0.57, 0.97)	1.13 (0.85, 1.50)	0.95 (0.77, 1.19)
$35000 to $69999	1.06 (0.78, 1.46)	0.98 (0.81, 1.18)	0.92 (0.74, 1.15)	1.29 (1.12, 1.48)	0.84 (0.69, 1.02)
$70000+	Reference	Reference	Reference	Reference	Reference
**Education**					
0 to 11 years (less than high school)	Reference	Reference	Reference	Reference	Reference
12 years (equivalent of high school)	1.50 (1.04, 2.16)	1.05 (0.84, 1.30)	1.36 (1.02, 1.82)	0.88 (0.69, 1.13)	1.09 (0.85, 1.40)
13 to 15 years (some college)	2.30 (1.64, 3.22)	1.24 (1.00, 1.52)	1.56 (1.15, 2.10)	0.99 (0.79, 1.24)	1.05 (0.83, 1.34)
16-plus years (college degree or more)	2.19 (1.50, 3.20)	0.89 (0.64, 1.24)	1.59 (1.18, 2.15)	0.84 (0.66, 1.07)	0.86 (0.66, 1.12)
**Current Employment**					
Employed	Reference	Reference	Reference	Reference	Reference
Unemployed	1.72 (0.98, 3.01)	0.79 (0.52, 1.19)	0.53 (0.35, 0.80)	0.78 (0.54, 1.13)	0.82 (0.56, 1.21)
Not in labor force	1.29 (0.93, 1.78)	1.44 (1.21, 1.71)	1.11 (0.90, 1.36)	1.13 (0.92, 1.39)	1.12 (0.91, 1.37)
**Marital Status**					
Married/Cohabitating	Reference	Reference	Reference	Reference	Reference
Divorced/Separated/Widowed	0.98 (0.71, 1.36)	1.06 (0.90, 1.25)	1.15 (0.96, 1.38)	1.41 (1.09, 1.81)	1.29 (1.06, 1.57)
Never Married	0.55 (0.38, 0.81)	0.86 (0.69, 1.07)	0.67 (0.53, 0.85)	0.80 (0.65, 0.98)	0.75 (0.62, 0.90)
**Insurance type**					
None	1.05 (0.65, 1.69)	1.06 (0.83, 1.36)	1.02 (0.77, 1.34)	1.36 (1.03, 1.81)	0.91 (0.74, 1.12)
Private	Reference	Reference	Reference	Reference	Reference
Public	1.55 (1.03, 2.31)	1.36 (0.97, 1.89)	1.26 (0.92, 1.72)	1.50 (1.20, 1.88)	1.12 (0.83, 1.50)

*All analyses applied appropriate sample weights.

† Odds ratios are adjusted for all other factors listed.

Table 3 shows the results of weighted adjusted analyses examining odds of mental health diagnoses across the 7-level rural-urban continuum variable. Aside from drug use disorders, a one-category increase in county rurality (as indicated by a 1-unit increase in the RUCC) was not associated with risk for overall lifetime psychiatric disorder. The odds of drug use disorder decreased with increasing rurality (aOR 0.89, 95% CI: 0.83–0.95).

**Table 3:** Adjusted odds ratios (aOR) for lifetime psychiatric disorder by rurality, NCS-R participants* †.

	Post-Traumatic Stress Disorder	Any Anxiety Disorder	Major Depressive Disorder	Any Mood Disorder	Any Impulse-Control Disorder	Alcohol Use Disorder	Drug Use Disorder	Any Substance Use Disorder	Any Disorder
	aOR (95% CI)	aOR (95% CI)	aOR (95% CI)	aOR (95% CI)	aOR (95% CI)	aOR (95% CI)	aOR (95% CI)	aOR (95% CI)	aOR (95% CI)
**Rurality** (1-unit increase)	1.00 (0.91, 1.11)	1.01 (0.92, 1.12)	1.01 (0.94, 1.09)	1.01 (0.94, 1.08)	0.97 (0.89, 1.06)	0.95 (0.88, 1.03)	0.89 (0.83, 0.95)	0.97 (0.88, 1.06)	0.99 (0.89, 1.10)
**Female** (vs. Male)	2.88 (2.35, 3.53)	1.78 (1.52, 2.08)	1.68 (1.46, 1.93)	1.67 (1.45, 1.91)	0.64 (0.54, 0.75)	0.31 (0.27, 0.36)	0.38 (0.31, 0.46)	0.44 (0.39, 0.51)	1.13 (0.96, 1.33)
**Race**									
Hispanic	0.63 (0.42, 0.95)	0.55 (0.43, 0.71)	0.70 (0.53, 0.92)	0.69 (0.53, 0.88)	0.62 (0.44, 0.86)	0.75 (0.56, 0.99)	0.64 (0.46, 0.87)	0.64 (0.49, 0.84)	0.52 (0.37, 0.73)
Non-Hispanic Black	0.79 (0.59, 1.08)	0.66 (0.54, 0.82)	0.49 (0.38, 0.64)	0.51 (0.40, 0.64)	0.65 (0.48, 0.89)	0.49 (0.38, 0.62)	0.48 (0.34, 0.69)	0.50 (0.38, 0.65)	0.60 (0.47, 0.76)
Non-Hispanic White	Reference	Reference	Reference	Reference	Reference	Reference	Reference	Reference	Reference
Other	0.97 (0.46, 2.03)	1.08 (0.71, 1.63)	0.98 (0.69, 1.39)	0.98 (0.67, 1.43)	1.06 (0.65, 1.73)	1.08 (0.69, 1.69)	1.02 (0.62, 1.69)	1.24 (0.78, 1.95)	0.95 (0.57, 1.59)
**Age**									
18 to 29	Reference	Reference	Reference	Reference	Reference	Reference	Reference	Reference	Reference
30 to 44	1.22 (0.86, 1.72)	1.09 (0.90, 1.33)	1.29 (1.05, 1.58)	1.28 (1.06, 1.54)	0.79 (0.65, 0.96)	1.32 (1.07, 1.65)	1.18 (0.87, 1.61)	1.22 (0.98, 1.51)	1.06 (0.83, 1.36)
45 to 59	1.28 (0.94, 1.74)	1.06 (0.82, 1.36)	1.25 (1.02, 1.53)	1.21 (1.00, 1.46)	0.17 (0.13, 0.22)	1.11 (0.86, 1.43)	0.61 (0.44, 0.84)	1.17 (0.93, 1.45)	0.84 (0.64, 1.09)
60+	0.21 (0.13, 0.33)	0.32 (0.24, 0.41)	0.52 (0.39, 0.69)	0.41 (0.31, 0.56)	0.05 (0.03, 0.08)	0.35 (0.25, 0.50)	0.02 (0.01, 0.05)	0.45 (0.33, 0.61)	0.28 (0.21, 0.36)
**Household Income**									
$0 to $19999	1.17 (0.80, 1.71)	0.98 (0.77, 1.24)	0.79 (0.64, 0.97)	0.87 (0.72, 1.04)	1.04 (0.81, 1.34)	1.49 (1.08, 2.05)	1.30 (0.91, 1.85)	1.38 (1.05, 1.82)	0.98 (0.78, 1.24)
$20000 to $34999	1.45 (0.87, 2.42)	1.29 (1.00, 1.67)	0.88 (0.73, 1.07)	0.97 (0.81, 1.16)	0.96 (0.71, 1.30)	1.35 (0.96, 1.89)	1.08 (0.73, 1.59)	1.16 (0.85, 1.59)	1.15 (0.86, 1.53)
$35000 to $69999	1.26 (0.97, 1.63)	1.10 (0.92, 1.33)	1.03 (0.87, 1.23)	1.08 (0.92, 1.26)	1.14 (0.91, 1.44)	1.54 (1.24, 1.90)	1.36 (1.03, 1.80)	1.36 (1.15, 1.60)	1.20 (0.98, 1.47)
$70000+	Reference	Reference	Reference	Reference	Reference	Reference	Reference	Reference	Reference
**Education**									
0 to 11 years (less than high school)	Reference	Reference	Reference	Reference	Reference	Reference	Reference	Reference	Reference
12 years (equivalent of high school)	0.55 (0.41, 0.73)	0.80 (0.62, 1.03)	1.07 (0.83, 1.37)	1.04 (0.83, 1.29)	0.67 (0.51, 0.89)	0.77 (0.54, 1.12)	0.80 (0.61, 1.04)	0.77 (0.57, 1.03)	0.81 (0.62, 1.07)
13 to 15 years (some college)	0.74 (0.53, 1.02)	0.81 (0.64, 1.04)	1.25 (1.04, 1.49)	1.20 (0.99, 1.45)	0.65 (0.48, 0.89)	0.72 (0.49, 1.05)	0.89 (0.64, 1.25)	0.78 (0.57, 1.07)	0.86 (0.64, 1.16)
16-plus years (college degree or more)	0.71 (0.47, 1.07)	0.80 (0.66, 0.98)	1.15 (0.92, 1.44)	1.07 (0.85, 1.35)	0.42 (0.29, 0.62)	0.55 (0.37, 0.81)	0.47 (0.34, 0.64)	0.54 (0.38, 0.77)	0.72 (0.56, 0.94)
**Current Employment**									
Employed	Reference	Reference	Reference	Reference	Reference	Reference	Reference	Reference	Reference
Unemployed	0.55 (0.29, 1.05)	1.07 (0.75, 1.54)	0.85 (0.63, 1.14)	0.95 (0.70, 1.30)	1.13 (0.72, 1.77)	0.78 (0.46, 1.30)	0.69 (0.31, 1.52)	0.72 (0.46, 1.12)	0.97 (0.67, 1.41)
Not in labor force	1.38 (1.09, 1.74)	1.07 (0.90, 1.27)	1.05 (0.88, 1.26)	1.18 (1.00, 1.38)	1.09 (0.89, 1.35)	1.02 (0.84, 1.23)	0.97 (0.76, 1.25)	1.09 (0.91, 1.30)	1.02 (0.84, 1.25)
**Marital Status**									
Married/Cohabitating	Reference	Reference	Reference	Reference	Reference	Reference	Reference	Reference	Reference
Divorced/Separated/Widowed	1.47 (1.06, 2.03)	1.23 (0.98, 1.55)	1.71 (1.42, 2.07)	1.68 (1.39, 2.02)	1.12 (0.90, 1.40)	1.55 (1.25, 1.93)	1.48 (1.10, 1.98)	1.58 (1.26, 1.99)	1.40 (1.11, 1.78)
Never Married	0.79 (0.55, 1.14)	1.01 (0.80, 1.27)	1.25 (1.00, 1.55)	1.18 (0.96, 1.45)	0.81 (0.65, 1.01)	1.16 (0.92, 1.46)	1.03 (0.79, 1.36)	1.12 (0.90, 1.38)	0.99 (0.77, 1.27)
**Insurance type**									
None	1.22 (0.95, 1.56)	1.27 (1.03, 1.57)	1.12 (0.91, 1.37)	1.11 (0.92, 1.32)	1.28 (0.98, 1.68)	1.68 (1.33, 2.14)	1.75 (1.31, 2.35)	1.59 (1.28, 1.97)	1.31 (1.03, 1.66)
Private	Reference	Reference	Reference	Reference	Reference	Reference	Reference	Reference	Reference
Public	1.90 (1.29, 2.78)	1.28 (0.98, 1.67)	1.32 (1.05, 1.65)	1.47 (1.15, 1.88)	1.53 (1.10, 2.13)	1.78 (1.41, 2.25)	2.37 (1.69, 3.32)	1.62 (1.27, 2.05)	1.29 (0.97, 1.73)

*All analyses applied appropriate sample weights.

† Odds ratios are adjusted for all other factors listed.

Other notable secondary findings with respect to our trauma exposure variables include the fact that individuals aged 60+ were three times more likely to have experienced war-related trauma compared to individuals less than 29 years of age (aOR = 3.35, 95% CI: 2.01–5.61). Females were overall less likely to experience most traumas compared to males, and individuals who were never married had decreased odds for most traumas compared to those who were married or cohabitating.

Other notable secondary findings with respect to our mental health disorders include that females had a more than two-folds greater odds of a lifetime history of PTSD compared to males (aOR = 2.88, 95% CI: 2.35–3.53) but were less likely to experience alcohol use disorders (aOR = 0.31, 95% CI: 0.27–0.36), drug use disorders (aOR = 0.38, 95% CI: 0.31–0.46) and any impulse control disorders (OR = 0.64, 95% CI: 0.54–0.75) compared to males (Table 3). Compared to whites, those who self-identified as Hispanic (aOR  = 0.52, 95% CI: 0.37–0.73), or non-Hispanic Black (aOR = 0.60, 95% CI: 0.47–0.76) were less likely to experience overall lifetime psychiatric disorders. Individuals who were divorced, separated or widowed were at a greater risk for most psychiatric disorders including PTSD, major depressive disorder, any mood disorder, alcohol use disorders, drug use disorders, and any substance use disorder compared to those who were married or cohabitating.

Of note, the results of our post-hoc analyses to examine whether threshold or nonlinear effects might account for our findings yielded similar results to the primary analyses presented above. When we substituted a 3-level rurality indicator (rural/suburban/major metropolitan) for the 7-level rurality variable, adjusting for other factors, we found similar results: compared to major metropolitan counties, suburban counties were less likely to report war-related trauma (aOR 0.55 95%CI: 0.32–0.95); while the findings for rural counties trended in the same direction but were not significant (aOR 0.65, 95%CI: 0.39–1.09). When we examined a threshold effect using a 2-level rurality indicator comparing major metropolitan counties to all other counties, we found that compared to major metropolitan counties, residents of all other counties were less likely to report war-related trauma (aOR 0.60, 95% CI: 0.41–0.89).

Similarly, we found little difference when we examined for threshold and nonlinear effects among psychiatric disorders. Using a using a 3-level rurality indicator, we found that residents of suburban counties trended towards lower drug use disorders (aOR 0.54, 95%CI: 0.29–1.01) and residents of rural counties had significantly lower drug use disorders (aOR 0.64, 95% CI: 0.52–0.79), compared to residents of major metropolitan areas. Using a 2-level rurality indicator, we found that compared to residents of major metropolitan areas, residents of all other areas were less likely to report drug use disorders (aOR: 0.59, 95% CI: 0.42–0.83). Similar to the results reported in Table 3, the remaining findings were not significant.

## Discussion

Examining a wide range of trauma exposures, the only urban-rural difference noted was that risk for war-related trauma decreased with rurality. This may be partially due to U.S. epidemiology, in which less than one third of the veteran population resides in rural areas [27]. In addition, the category of war-related trauma also includes non-military trauma such as those experienced by refugees. A growing number of refugees world-wide are seeking protection in urban areas [28], and thus urban-residing refugees of foreign wars may also partially account for this finding.

The remaining trauma exposure categories were not significantly associated with position on the urban-rural continuum. Our finding that trauma exposures are relatively constant across the rural-urban continuum is unique in the literature and particularly important in light of data suggesting that trauma is under recognized in healthcare settings [29]. This finding is also notable as some rural primary care physicians (PCP) perceive trauma exposure to be an uncommon occurrence in their communities [14], because it suggests that rural patients may have even an even proportionally higher risk of their trauma histories going unrecognized by their healthcare providers compared to non-rural patients.

In our study, residents of metropolitan areas were more likely to report drug use disorders compared to residents of rural areas. This data is consistent with a report from the National Household Survey on Drug Abuse (NHSDA), which showed that individuals who lived in metropolitan areas are more likely to have used an illicit drug compared to nonmetropolitan residents [30]. Overall, other than a decreased prevalence of drug use disorder with increasing rurality, our study did not find any significant difference regarding lifetime psychiatric disorders based on position on the rural-urban continuum. This finding adds to the body of knowledge showing that rural residing persons are just as likely to have mental health diagnoses when compared to their urban counterparts [20], [21]. Moreover, we have shown this across a larger number of psychiatric disorders compared to what has been previously reported [12], [20]–[21] and in a national dataset unlike most previous studies [15]–[19].

Our findings are also interesting in light of recent national data from Breslau et al. [23] showing slightly elevated risk for major depression and serious mental illnesses in categories of intermediate rurality. It should be noted that Breslau, et al. used a novel indicator of rurality for their study, and the granularity this allowed in urban-rural classification may have accounted for the differences between our findings and that of Breslau and colleagues. Our post-hoc examinations of threshold and nonlinear effects did not produce substantially different findings compared to the primary analyses shown in Tables 2 and 3. However, future research should examine in more detail the possibility of a nonlinear association between rurality and mental health outcomes.

Rural communities suffer from a shortage of mental healthcare resources [31]. Mental health service providers tend to concentrate in urban areas and few rural areas are sufficiently served [32]. Additionally, residents of rural communities may have problems accessing mental health services, due to numerous factors including long distances to travel for mental healthcare, limited public transportation in rural areas and lack of mental health outreach [33]. Considering that the frequencies of lifetime psychiatric disorders are equivalent among rural and urban communities, our data strongly suggest a relative mental health service deficit for rural residents, which is likely to be exacerbated by underrecognition of trauma exposure in rural areas.

We also had several secondary findings worth examining. For example, although females in our sample were overall less likely to experience most of the traumas measured, females were more likely to report a lifetime history PTSD. This likely reflects two factors. First, there is a gender disparity in types of trauma to which individuals are exposed. The traumas measured in this study may reflect traumas that disproportionately affect men. For example, the traumas examined may not have included the full range of childhood traumas that have been shown to affect adult functioning, such as in the Adverse Childhood Experiences (ACE) Study [34]. Additionally, all domains of intimate partner violence, which disproportionately affects women, including emotional abuse [35] were not measured. If these traumas were examined, our findings may have been different with respect to gender and trauma frequency. Second, women who survive trauma are more susceptible to the development of PTSD than men who survive trauma [36]. Thus, even with the lower frequency of trauma exposures, the increased frequency of PTSD among women may be due to greater susceptibility to the development of PTSD among women.

Our findings with respect to race and ethnicity mirror those of other studies in the National Institute of Mental Health Collaborate Psychiatric Epidemiology Surveys (CPES), [37] which includes the NSC-R. In these studies, racial and ethnic minorities have been generally found to have lower rates of lifetime mental disorders compared to white, non-Hispanic participants [38]. We also found slightly reduced frequencies of accident-related trauma among Hispanic and non-Hispanic Black individuals compared to whites. This finding is intriguing and bears further investigation. However, available data suggest that when accidental injury occurs, as in the case of motor vehicle crashes, Blacks may be more likely to experience a fatality [39]. Our study also showed that markers of higher socioeconomic status (SES), such as higher educational attainment and greater education, are generally protective against substance use disorders. This is supported by the literature which shows that higher SES is generally protective against both initiation of substance use and also promotes cessation of substance use [40].

### Strengths and limitations

One of the main strengths of this study was the use of a nationally representative survey to assess both the prevalence of lifetime psychiatric disorders and to examine trauma exposure frequencies across the rural-urban continuum. In particular, the exploration of trauma exposure across the rural-urban continuum is, to our knowledge, novel in the literature.

Our study has several limitations to consider. We were unable to assess data from RUCC 8-9, the most rural counties, as individuals from these counties were not included in the NSC-R Part II survey sampling frame. Moreover, although the list of traumas examined was extensive, it was by no means exhaustive, which may have accounted for some of our findings with respect to gender.

Finally, we examined lifetime trauma exposures and mental health diagnoses and related them to position on the urban-rural continuum at the time of the study. Although this methodology is appropriate for informing allocation of healthcare services across the rural-urban continuum, it does not provide epidemiologic information regarding the residence of the subject at the time of the trauma exposure or mental health diagnosis. It is possible that some subjects migrated between rural and urban contexts between the time of their trauma exposure or development of a mental health diagnosis and the time of data collection. Future research should assess for the role of migration across the urban-rural continuum to better understand where trauma exposures first occurred and where mental health diagnoses were first identified. This may be particularly relevant for those for whom trauma exposures and mental health diagnoses occurred during childhood. Further study could examine recent mental health diagnoses in addition to lifetime diagnoses, to better inform public health officials regarding current need for mental health services across the rural-urban continuum.

## Conclusions

Across the rural-urban continuum, the frequencies of most lifetime psychiatric disorders and trauma exposures are similar. The only notable differences were that war-related traumas and drug use disorders were more prevalent in metropolitan areas. Given that rural communities suffer from a shortage of mental healthcare resources with a similar frequency of psychiatric disorders and traumatic exposures, our data suggests a substantial service deficit exists to adequately address the mental health needs of rural-residing individuals, and reinforces calls for improving access to mental health care in rural communities [41]. Given the high prevalence of both mental health disorders and trauma exposures across the rural-urban continuum, policies should promote education of both rural and nonrural healthcare providers to improve care of patients with mental illnesses and to provide trauma-informed care [42].

### Ethical approvals

Prior to subject involvement in the NCS-R, the human subject participant committees from both the Harvard Medical School and the University of Michigan provided ethical review for the parent study. The current report is a secondary analysis of NCS-R data. The Pennsylvania State University College of Medicine Institutional Review Board (IRB) reviewed and approved this secondary analysis.

## References

[1] Kessler RC, Wang PS (2008) The descriptive epidemiology of commonly occurring mental disorders in the United States. Annu Rev Public Health 29: 115–129. 18348707 10.1146/annurev.publhealth.29.020907.090847

[2] Insel TR (2008) Assessing the economic costs of serious mental illness. Am J Psychiatry 165: 663–665. 18519528 10.1176/appi.ajp.2008.08030366

[3] Kendler KS, Eaves LJ (1986) Models for the joint effect of genotype and environment on liability to psychiatric illness. Am J Psychiatry 143: 279–289. 3953861 10.1176/ajp.143.3.279

[4] Mcquaid JR, Pedrelli P, McCahill ME, Stein MB (2001) Reported trauma, post-traumatic stress disorder and major depression among primary care patients. Psychol Med 31: 1249–1257. 11681551 10.1017/s0033291701004202

[5] Kessler RC, Sonnega A, Bromet E, Hughes M, Nelson CB (1995) Posttraumatic stress disorder in the National Comorbidity Survey. Arch Gen Psychiatry 52: 1048–1060. 7492257 10.1001/archpsyc.1995.03950240066012

[6] Breslau N, Davis GC, Andreski P, Peterson E (1991) Traumatic events and posttraumatic stress disorder in an urban population of young adults. Arch Gen Psychiatry 48: 216–222. 1996917 10.1001/archpsyc.1991.01810270028003

[7] Hinton WL, Tiet Q, Tran CG, Chesney M (1997) Predictors of depression among refugees from Vietnam: a longitudinal study of new arrivals. J Nerv Ment Dis 185: 39–45. 9040532 10.1097/00005053-199701000-00007

[8] Rytwinski NK, Scur MD, Feeny NC, Youngstrom EA (2013) The co-occurrence of major depressive disorder among individuals with posttraumatic stress disorder: a meta-analysis. J Trauma Stress 26: 299–309. 23696449 10.1002/jts.21814

[9] Jacobsen LK, Southwick SM, Kosten TR (2001) Substance use disorders in patients with posttraumatic stress disorder: a review of the literature. Am J Psychiatry 158: 1184–1190. 11481147 10.1176/appi.ajp.158.8.1184

[10] Ricketts TC, Johnson-Webb KD, Randolph RK (1999) Populations and Places in Rural America. In: Ricketts TC, editor. Rural health in the United States. New York: Oxford University Press. pp. 7–24.

[11] Schur C, Franco S (1999) Access to health care. In: Ricketts TC, editor. Rural Health in the United States. New York: Oxford University Press. pp. 25–37.

[12] Probst JC, Laditka SB, Moore CG, Harun N, Powell MP, et al (2006) Rural-urban differences in depression prevalence: implications for family medicine. Fam Med 38: 653–660. 17009190

[13] Gamm LD, Hutchison LL, Dabney BJ, Dorsey AM (2003) Rural Healthy People 2010: A Companion Document to Healthy People 2010. College Station, Texas: The Texas A&M University Health Science Center, School of Rural Public Health, Southwest Rural Health Research Center.

[14] Colon-Gonzalez MC, McCall-Hosenfeld JS, Weisman CS, Hillemeier MM, Perry AN, et al (2013) ‘Someone’s got to do it' – Primary care providers (PCPs) describe caring for rural women with mental health problems. Mental Health in Family Medicine 10(4): 191. 25632302 PMC4306261

[15] Vega WA, Kolody B, Aguilar-Gaxiola S, Alderete E, Catalano R, et al (1998) Lifetime prevalence of DSM-III-R psychiatric disorders among urban and rural Mexican Americans in California. Arch Gen Psychiatry 55: 771–778. 9736002 10.1001/archpsyc.55.9.771

[16] Burris JL, Andrykowski M (2010) Disparities in mental health between rural and nonrural cancer survivors: a preliminary study. Psychooncology 19: 637–645. 19582800 10.1002/pon.1600 PMC2880195

[17] Blazer D, George LK, Landerman R, Pennybacker M, Melville ML, et al (1985) Psychiatric disorders. A rural/urban comparison. Arch Gen Psychiatry 42: 651–656. 4015306 10.1001/archpsyc.1985.01790300013002

[18] Rueter MA, Holm KE, Burzette R, Kim KJ, Conger RD (2007) Mental health of rural young adults: prevalence of psychiatric disorders, comorbidity, and service utilization. Community Ment Health J 43: 229–249. 17345147 10.1007/s10597-007-9082-y

[19] Philbrick JT, Connelly JE, Wofford AB (1996) The prevalence of mental disorders in rural office practice. J Gen Intern Med 11: 9–15. 8691295 10.1007/BF02603478

[20] Blazer DG, Kessler RC, McGonagle KA, Swartz MS (1994) The prevalence and distribution of major depression in a national community sample: the National Comorbidity Survey. Am J Psychiatry 151: 979–986. 8010383 10.1176/ajp.151.7.979

[21] Kessler RC, Berglund P, Demler O, Jin R, Koretz D, et al (2003) The epidemiology of major depressive disorder: results from the National Comorbidity Survey Replication (NCS-R). JAMA 289: 3095–3105. 12813115 10.1001/jama.289.23.3095

[22] Peen J, Schoevers RA, Beekman AT, Dekker J (2009) The current status of urban-rural differences in psychiatric disorders. Acta Psychiatr Scand 121: 84–93. 19624573 10.1111/j.1600-0447.2009.01438.x

[23] Breslau J, Marshall GN, Pincus HA, Brown RA (2014) Are mental disorders more common in urban than rural areas of the United States? 10.1016/j.jpsychires.2014.05.004 24857610

[24] Kessler RC, Berglund P, Chiu WT, Demler O, Heeringa S, et al (2004) The US National Comorbidity Survey Replication (NCS-R): design and field procedures. Int J Methods Psychiatr Res 13: 69–92. 15297905 10.1002/mpr.167 PMC6878537

[25] United States Department of Agriculture (2003) USDA-ERS - Rural Classifications. [Internet]. [Updated 7/29/2004, cited 7/17/2014]. Available from: http://www.ers.usda.gov/Briefing/Rurality/RuralUrbCon/.

[26] Nickerson A, Aderka IM, Bryant RA, Hofmann SG (2012) The relationship between childhood exposure to trauma and intermittent explosive disorder. Psychiatry Res 197: 128–134. 22464047 10.1016/j.psychres.2012.01.012

[27] ORH: VHA Office of Rural Health (2014) Fact Sheet. Information about the VHA Office of Rural health and Rural Veterans. [Internet]. ORH: VHA Office of Rural Health. [Updated May 2014; cited 7/17/2014] Available from: http://www.ruralhealth.va.gov/docs/factsheets/ORH_General_FactSheet_2014.pdf

[28] UN High Commissioner for Refugees (UNHCR) (2009) UNHCR policy on refugee protection and solutions in urban areas [Internet]. (Cited 9/11/2014). Available from: www.refworld/org/docid/4ab8e7f72.html.

[29] Samson AY, Bensen S, Beck A, Price D, Nimmer C (1999) Posttraumatic stress disorder in primary care. J Fam Pract 48: 222–227. 10086767

[30] National Household Survey on Drug Abuse (2002) Illicit drug use in Metropolitan and Non-Metropolitan areas. [Internet] [Updated August 2, 2002, cited 7/17/2014]. Available from: http://www.samhsa.gov/data/2k2/Urban/Urban.pdf.

[31] Merwin E, Hinton I, Dembling B, Stern S (2003) Shortages of rural mental health professionals. Arch Psychiatr Nurs 17: 42–51. 12642887 10.1053/apnu.2003.1

[32] Murray JD, Keller PA (1991) Psychology and rural America. Current status and future directions. Am Psychol 46: 220–231. 2035932 10.1037//0003-066x.46.3.220

[33] Human J, Wasem C (1991) Rural mental health in America. Am Psychol 46: 232–239. 2035933 10.1037//0003-066x.46.3.232

[34] Felliti VJ, Anda RF, Nordenberg D, Williamson DF, Spitz AM, et al (1998) Relationship of Childhood Abuse and Household Dysfunction to Many of the Leading Causes of Death in Adults: The Adverse Childhood Experiences (ACE) Study. Am J Prev Med 14(4): 245–258. 9635069 10.1016/s0749-3797(98)00017-8

[35] CDC (2012) “Understanding Intimate Partner Violence: Fact Sheet, 2012” [Internet]. (Cited 9/11/2014) Available from: www.cdc/gov/violenceprevention/pdf/ipv_factsheet2102-a.pdf.

[36] Stein MB, Walker JR, Forde DR (2000) Gender differences in susceptibility to posttraumatic stress disorder. Behav Res Ther 38(6): 619–28. 10846810 10.1016/s0005-7967(99)00098-4

[37] Collaborativee Psychiatric Epidemiology Surveys (CPES) Welcome to CPES. [Internet] (Cited 09/20/2014). Available from: www.icpsr.umich.edu/icsprweb/CPES

[38] McGuire TG, Miranda J (2008) Racial and ethnic disparities in mental health care: evidence and policy implications. Health Aff (Milwood) 27(2): 393–403. 10.1377/hlthaff.27.2.393 PMC3928067 18332495

[39] West BA, Naumann RB (2011) Motor vehicle-related deaths – United States, 2003–2007. MMWR 60 (Supplement): 52–55. 21430621

[40] Galea S, Nandi A, Vlahov D (2004) The Social Epidemiology of Substance Use. Epidemiol Rev 26(1): 36–52 doi: 10.1093/epirev/mxh007 15234946 10.1093/epirev/mxh007

[41] President's New Freedom Commission on Mental Health (2003) Achieving the Promise: Transforming Mental Healthcare in America. [Internet]. [Updated July 22, 2003, Cited 7/7/2014]. Available from: http://govinfo.library.unt.edu/mentalhealthcommission/reports/FinalReport/downloads/FinalReport.pdf.

[42] The National Center for Trauma-Informed Care [Internet]. (Cited 7/7/2014). Available from: http://www.nasmhpd.org/TA/nctic.aspx.

